# Rapid Prototyping of Nanofluidic Slits in a Silicone Bilayer

**DOI:** 10.6028/jres.120.015

**Published:** 2015-11-17

**Authors:** Thomas P. Kole, Kuo-Tang Liao, Daniel Schiffels, B. Robert Ilic, Elizabeth A. Strychalski, Jason G. Kralj, J. Alexander Liddle, Anatoly Dritschilo, Samuel M. Stavis

**Affiliations:** 1 National Institute of Standards and Technology, Gaithersburg, MD 20899; 2 MedStar Georgetown University Hospital, Department of Radiation Medicine, Washington, DC 20007; 3 University of Maryland, Maryland Nanocenter, College Park, MD 20740

**Keywords:** device, DNA, microfluidic, molding, nanofluidic, nanoparticle, silicone, prototyping, replica, silicon, silicone, SU-8^1^

## Abstract

This article reports a process for rapidly prototyping nanofluidic devices, particularly those comprising slits with microscale widths and nanoscale depths, in silicone. This process consists of designing a nanofluidic device, fabricating a photomask, fabricating a device mold in epoxy photoresist, molding a device in silicone, cutting and punching a molded silicone device, bonding a silicone device to a glass substrate, and filling the device with aqueous solution. By using a bilayer of hard and soft silicone, we have formed and filled nanofluidic slits with depths of less than 400 nm and aspect ratios of width to depth exceeding 250 without collapse of the slits. An important attribute of this article is that the description of this rapid prototyping process is very comprehensive, presenting context and details which are highly relevant to the rational implementation and reliable repetition of the process. Moreover, this process makes use of equipment commonly found in nanofabrication facilities and research laboratories, facilitating the broad adaptation and application of the process. Therefore, while this article specifically informs users of the Center for Nanoscale Science and Technology (CNST) at the National Institute of Standards and Technology (NIST), we anticipate that this information will be generally useful for the nanofabrication and nanofluidics research communities at large, and particularly useful for neophyte nanofabricators and nanofluidicists.

## 1. Introduction

Nanofluidic devices comprise enclosed channels to confine materials and control interactions in fluids and near solid–fluid interfaces at characteristic submicrometer length scales. In this way, nanofluidic devices are useful for the manipulation and measurement of nanoscale materials, such as biomolecules and nanoparticles, and related nanoscale interactions, including steric, electrostatic, hydrodynamic, and entropic effects. As such, nanofluidic devices have found use in diverse applications in the biological, chemical, and physical sciences involving such materials and interactions [1].

**Fig. 1:**
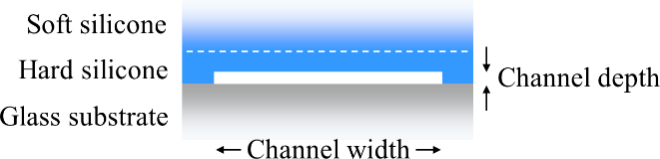
Schematic illustrating a nanofluidic channel with a high aspect ratio of width to depth, in cross section, not to scale. The nanoscale depth of this nanofluidic slit corresponds to the characteristic dimension of a material or interaction of interest. A thin film of hard silicone accepts the slit pattern and prevents the microscopic collapse of the channel. A thick layer of soft silicone supports the thin film of hard silicone and maintains macroscopic compliance of the silicone bilayer for handling and bonding.

Nanofabrication is foundational to nanofluidics, enabling implementation of the structural geometries and material properties of nanofluidic devices for these diverse applications. Lithographic processes, in particular, facilitate the deterministic fabrication of nanofluidic devices [2]. In the early years of nanofluidics, patterning and etching nanofluidic channels into a substrate, and then bonding a cover to the substrate, was a common process for fabricating nanofluidic devices. Silicon and various forms of silicon dioxide are typical hard materials for substrates and covers. Nanofluidic devices fabricated in this way are mechanically rigid and chemically stable, which is advantageous for many applications. Silicon dioxide, in addition, is electrically insulating and optically transparent, with low levels of autofluorescence [3]. This type of fabrication process is still useful, but in recent years, a trend has emerged of fabricating nanofluidic devices in soft materials to capitalize on the advantages of soft lithography [4]. These advantages include short cycles of designing, fabricating, and testing prototype devices, the ability to process organic materials and flexible substrates that are not compatible with standard processes for manufacturing integrated circuits, and the economy of devices replicated in plastics. While this trend has occurred primarily in the context of research and development, it is also relevant to the commercial manufacture of nanofluidic devices at low marginal cost. The molding of microfluidic devices in soft materials such as polydimethylsiloxane (PDMS), also known as silicone, has become commonplace, as this material can be optically transparent, biologically compatible, and gas permeable, making it suitable for culturing living cells with microscale dimensions. Closely related techniques can also apply to fabricate nanofluidic devices [1]. There are, however, many process parameters that must be under control to reliably fabricate fluidic devices in soft materials at the nanometer scale, particularly channels with high aspect ratios of channel width to channel depth, as Fig. 1 illustrates. Such nanofluidic slits are useful for many applications, as Sec. 2 discusses, but are prone to collapse due to mechanical flexibility [5]. This motivates the development of a comprehensive and accessible process for rapidly prototyping nanofluidic slits in silicone, while maintaining the structural integrity of the devices. This article reports such a process.

The rapid prototyping process that this article reports builds on a large body of related literature. An important distinction of this article is that the description of this process is very comprehensive, presenting context and details that previous articles reporting on related topics have not discussed. This information is highly relevant to the rational implementation and reliable repetition of the process. We developed this process at the Center for Nanoscale Science and Technology (CNST) at the National Institute of Standards and Technology (NIST), using certain commercial equipment, instruments, and materials. Other nanofabrication facilities and research laboratories have comparable equipment, instruments, and materials from various commercial vendors, to which this process is potentially adaptable. Therefore, while this article specifically informs users of the CNST, we anticipate that this information will be generally useful for the nanofabrication and nanofluidics research communities at large, and particularly useful to neophyte nanofabricators and nanofluidicists.

## 2. Designing a Device

**Fig. 2:**
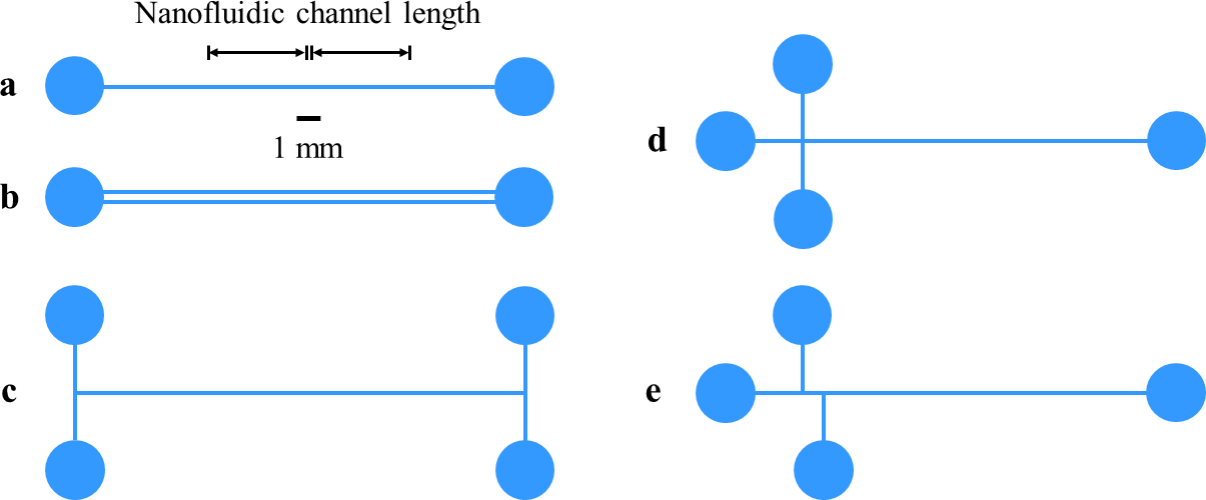
Schematics illustrating the layout of exemplary nanofluidic devices, from the top down, approximately to scale. The length of a nanofluidic channel typically varies from 0.1 mm to 10 mm, although shorter or longer nanofluidic channels can also be useful, depending on the intended function of the device. Microfluidic channels often connect the nanofluidic channel to inlet and outlet reservoirs. (a) Schematic showing a simple device with a single channel, inlet, and outlet. (b) Schematic showing a device with parallel channels that share a common inlet and outlet. (c) Schematic showing a device with an H–junction. (d) Schematic showing a device with an X–junction. (e) Schematic showing a device with a double–T–junction.

A variety of factors constrain the design of a nanofluidic device, including the critical dimensions and resulting functionality, overall layout and laboratory interface, and the process used to fabricate the device. First and foremost, certain dimensions of the device, typically the depth and possibly the width of the primary channel, as Fig. 1 illustrates, are critical to implementing the intended functionality of the device. The length of the primary channel can also be a critical dimension. However, the overall layout of the device frequently influences this dimension, as Fig. 2 illustrates, more so than the other dimensions of the primary channel. In this regard, the interface of the device with laboratory equipment for sensing and actuating nanoscale materials and interactions within the device, such as an optical microscope or an electrode in a fluid reservoir, is an important consideration in the design process. Last but not least, the fabrication process imposes constraints on device design, including the maximum aspect ratio of slit width to depth and the overall layout of the device. While the particular design of any nanofluidic device depends on the specific application of the user, some general considerations for device design are as follows.

For rigid nanofluidic slits, the depth or depths of the primary channel correspond to the characteristic dimensions of the materials or interactions in confinement, determining the nanofluidic functionality. For example, as the depth of a nanofluidic slit decreases through the submicrometer scale and into the nanometer scale, the top and bottom surfaces of the slit confine long duplex DNA molecules in the vertical direction, and extend the DNA molecules in the lateral dimensions [6, 7]. Nanofluidic slits with depths of less than twice the hydrodynamic radius of nanoparticles, exclude nanoparticles by size from these shallower regions of the slits [8]. In these examples, interfaces between nanofluidic slits of different depths provide additional nanofluidic functionality, resulting in the application of entropic forces to DNA molecules [9], and providing resolution and range in the separation and characterization of nanoparticles [10]. In an electrolyte solution with a low ionic strength, the screening distance of electrostatic interactions can extend into the submicrometer scale, and thus can overlap between the top and bottom surfaces of a nanofluidic slit, resulting in concentration polarization [11]. Beyond these exemplary materials and interactions within a nanofluidic slit, the depth of a nanofluidic slit can correspond to external factors relating to laboratory equipment, such as the depth of field of an optical microscope, so that all objects confined to the slit are in focus.

The width of the nanofluidic channel can also be functional. The side walls of a channel impose additional confinement and can result in electrostatic interactions at the corners [12]. The width of a nanofluidic slit can be less than the field of view of an optical microscope, so that all objects within this region of the slit are in view.

The length of the nanofluidic channel determines the spatial gradient of an applied pressure or voltage, resulting in a flow field or electric field which can transport nanoscale materials through the channel and separate the constituents of a mixture of nanoscale materials by differential mobility. Practical limitations associated with the total area of the device and placement of fluid reservoirs also influence the length of the nanofluidic channel. This length is often only as long as is necessary to achieve the intended nanofluidic functionality, with microfluidic channels connecting this functional length of the device to inlet and outlet reservoirs. In comparison to nanofluidic slits, microfluidic channels are more robust against fabrication defects, are less prone to clogging, and result in lower field gradients in these connecting regions of the device. Section 4 describes related aspects of device fabrication.

In addition to these practical issues, the interface between nanofluidic and microfluidic channels can be functional, or dysfunctional, depending on the application. For example, different manipulations of the same nanoscale material, long duplex DNA, impose opposing constraints on the design of this interface. By default, planar photolithography typically results in a stepped interface. The resulting abrupt change in the free energy of confinement can be useful for the entropic separation of DNA molecules of different lengths [13]. In contrast, for ease of loading long duplex DNA into strong confinement, it can be useful to have a gradually varying depth and free energy of confinement [14–16]. It is possible to fabricate such structures with photolithography, but this is beyond the scope of this article.

The intended functionality of a nanofluidic device influences the overall layout of the device. The simplest layout of a nanofluidic device is a single channel with one inlet reservoir and one outlet reservoir, as Fig. 2(a) shows. This layout is useful for the confinement and transport of nanoscale materials. Multiple channels can form a parallel array, with common inlets and outlets, as Fig. 2(b) shows, or with independent inlets and outlets. This layout enables the parallel analysis of multiple channels. An H–junction, as Fig. 2(c) shows, provides more control over sample loading and electrokinetic drive [17]. An X–junction, as Fig. 2(d) shows, allows the formation of a small sample volume for subsequent separation by differential mobility of the constituents of the mixture. A double–T–junction [18], as Fig. 2(e) shows, allows better control over the formation of this sample volume for separation. The laboratory interface and fabrication process also influence the device layout, as follows.

The overall layout of the device must accommodate the interface of the device with external laboratory equipment, in particular any optical microscope used for imaging and tracking nanoscale materials within the device. The nanofluidic device must physically interface with the microscope objective lens. For sensitive detection of faint signals, this lens often has a magnification > 60×, a numerical aperture > 1, and optical corrections for imaging through a No. 1½ glass coverslip with a thickness of ≈ 0.17 mm. Reservoirs, tubing, capillaries, electrodes, and the like must be clear of the interface between nanofluidic device and objective lens. The nanofluidic channel must also interface with the inlet and outlet reservoirs, often through microfluidic channels. For a transparent substrate, such as a microscope coverslip, the reservoirs must not extend over the region of the nanofluidic device to be imaged. This constraint typically sets a minimum distance of a few centimeters between the inlet and outlet reservoirs.

Finally, the rapid prototyping process that this article reports introduces several constraints on device design. In many research facilities, a silicon wafer with a diameter of 100 mm and a thickness of ≈ 500 μm is a standard substrate upon which to pattern the inverse features of fluidic devices for molding of soft materials. The process of manually spin-coating photoresist over such a wafer, using typical equipment found in a research facility, can result in radial variation in film thickness. This variation can become significant, if spin-coating photoresist coated over previously patterned features that are comparable in height to the thickness of the film, which may occur in a multilevel photolithography process. Since the thickness of the photoresist film determines the depth of the molded channels in the rapid prototyping process that this article reports, this potential issue is a relevant consideration in the process of designing a device, to avoid the need for a planarization process. In addition, in a multilevel photolithography process, the device design must incorporate alignment marks. The following discussion of device design addresses these issues, among others.

### 2.1

Consider the relation between the design of the nanofluidic device and the substrate upon which inverse features are patterned to form the device mold.

#### 2.1.1

Typically, a wafer with a diameter of 100 mm can accommodate an array of more than one device mold, increasing the yield of the fabrication process. The maximum number of device molds in an array depends on the size of each device, and the number and location of inlet and outlet reservoirs.

#### 2.1.2

Simultaneously, an optimal array of device molds minimizes the impact of variation in the dimensions of the devices across a wafer due to the fabrication process. Sec. 2.3 discusses this issue in more detail.

#### 2.1.3

Defects at the wafer edge occurring during the fabrication process, such as an edge bead or handling mark in the photoresist film, can compromise device molds close to the edge of the substrate. Ten millimeters is a conservative margin to avoid such defects, defining the periphery of the array.

### 2.2

Use computer aided design (CAD) software to layout the device. Use the outline of a wafer with a diameter of 100 mm and a standard flat as the furthest outer extent of the array of devices, and pattern device features within the center ≈ 90 mm of the wafer to avoid possible edge defects.

### 2.3

For devices fabricated with a single level of lithography and a single photomask, place every feature on a single design level, with no alignment marks. For devices fabricated with multiple levels of lithography, requiring spin-coating of photoresist over patterned features and alignment between subsequent levels of lithography, observe the following guidelines.

#### 2.3.1

**Fig. 3:**
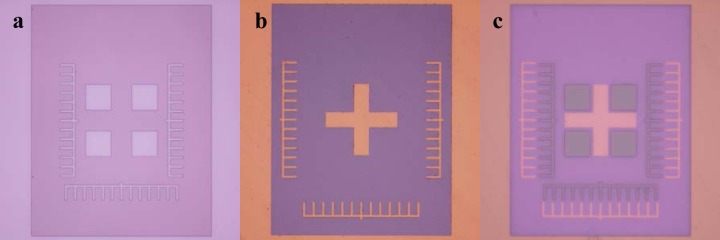
(a) Optical micrograph showing a lower alignment mark patterned on a substrate in the first level of photolithography. (b) Optical micrograph showing an upper alignment mark patterned on a photomask for the second level of photolithography. (c) Optical micrograph showing the alignment of the upper alignment mark through the photomask to the lower alignment mark on the substrate during the second level of photolithography.

Place device features, such as nanofluidic slits, and lower alignment marks for the substrate, as Fig. 3(a) shows, on the first design level. This order is important because it is optimal to pattern features with nanoscale vertical dimensions in a planar film of photoresist, coated conformally over an unpatterned substrate. If the vertical dimensions of features patterned in subsequent levels of photolithography are critical, and if the designs of the device and array permit, then place features on lower levels towards the periphery of the array. This initial placement will reduce variation in film thickness from spin-coating photoresist over these lower-level features, at locations nearer the center of the wafer where higher-level features will subsequently be placed.

#### 2.3.2

Place lower alignment marks towards the periphery of the wafer to improve angular alignment and to reduce resulting radial variation in film thickness, and in locations that do not interfere with device features. An ideal placement of lower alignment marks allows the use of a single, linear strip of tape to mask multiple lower alignment marks during spin-coating of photoresist, as Sec. 4.8 describes, without covering any interstitial device features. This preserves the visibility of the lower alignment marks while performing higher levels of photolithography. Include at least one pair of lower alignment marks for each higher level of photolithography.

#### 2.3.3

**Fig. 4:**
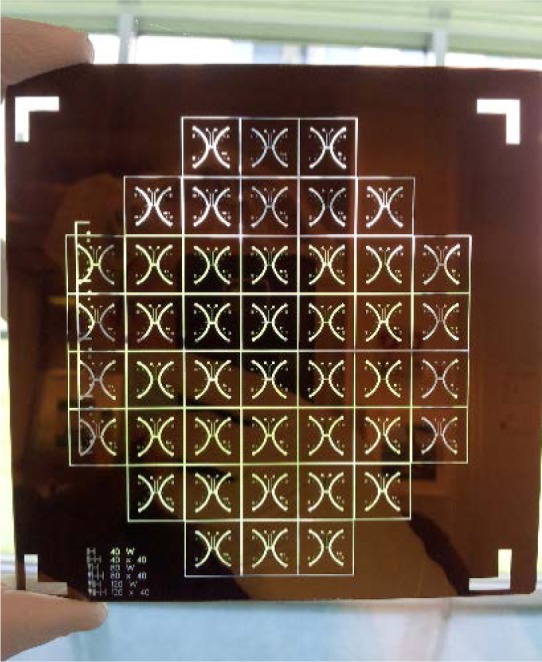
Photograph showing a photomask with a square size of 127 mm (5 in) with patterned arrays of channels, inlets, and outlets, visible as transparent glass features on an opaque chromium field for exposing a negative-tone photoresist. This is an aggressive design for a device array, in that the outermost patterned features will approach the edge of a wafer with a diameter of 100 mm, where defects become possible. The hand in the photograph is gloved, reducing contamination of photomasks and wafers.

Place device features such as microfluidic channels, inlet reservoirs, and outlet reservoirs, and upper alignment marks for the photomask, as Fig. 3(b) shows, on higher design levels. If the vertical dimensions of these features are critical, and if the designs of the device and array permit, then place these higher-level features closer to the center of the wafer. This placement will benefit from the reduced variation in film thickness from spin-coating photoresist over the lower level features, which, as Sec. 2.3.1 describes, are optimally placed towards the periphery of the array. Lower alignment marks on the substrate must be visible through higher alignment marks on the photomask, which will be mostly opaque for patterning a mold structure in a negative-tone photoresist, as Fig. 4 shows.

#### 2.3.4

During the photolithography process, align the complementary alignment marks through the photomask to the substrate, as Fig. 3(c) shows.

## 3. Fabricating a Photomask

Photolithographic processes can form device patterns in thin films of photoresist, with lateral dimensions extending from the centimeter scale into the submicrometer scale. Through various processes of pattern transfer, thin films of photoresist can then form the vertical dimensions of these structures, extending from the micrometer scale into the nanometer scale. The potentially excellent control over the thickness of a film of photoresist can transfer directly to the depth of a molded channel, as Sec. 4 discusses. While photomasks are available for purchase from commercial vendors, this section describes how to rapidly fabricate a photomask in a clean room. Figure 4 shows a representative photomask for exposing a negative-tone photoresist. Table 1 presents materials for fabricating a photomask.

### 3.1

Expose the photomask using the Heidelberg Instruments DWL 2000 Laser Pattern Generator, with a laser wavelength of 405 nm, an address grid of 20 nm, and a writehead focal length of 4 mm. The exposure time changes as the laser ages, and the CNST NanoFab staff calibrates this parameter.

### 3.2

Immerse the photomask in AZ 400K developer 1:3 for approximately 60 s to develop the exposed pattern in the film of photoresist on the photomask. Rinse the photomask in deionized water and blow-dry with nitrogen.

### 3.3

Immerse the photomask in Chromium Etchant 1020 for approximately 60 s to transfer the pattern from the film of photoresist to the film of chromium on the photomask. Rinse the photomask in deionized water and blow-dry with nitrogen.

### 3.4

Inspect the photomask by optical microscopy and ensure that the chromium has etched completely. If any chromium residue persists, then immerse the photomask in Chromium Etchant 1020 for 10 s, rinse with deionized water, and blow-dry with nitrogen. Repeat as necessary.

### 3.5

Immerse the photomask in Remover PG for 10 min at room temperature with gentle agitation to strip the film of photoresist from the photomask. Rinse the photomask in deionized water and blow-dry with nitrogen.

### 3.6

Immerse the photomask in Nanostrip for 10 min at room temperature with gentle agitation to clean any remaining photoresist from the photomask. Rinse the photomask in deionized water and blow-dry with nitrogen.

## 4. Patterning a Device Mold

**Fig. 5:**
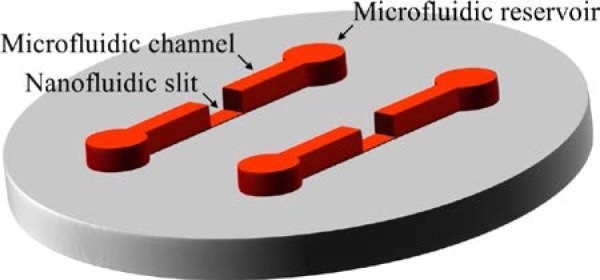
Schematic illustrating an array of device molds patterned in a bilayer of SU-8 on a silicon wafer, not to scale. A nanoscale film of SU-8 2000.5 forms the mold for nanofluidic slits. A microscale film of SU-8 2025 forms the mold for microfluidic channels and reservoirs.

There are many possible approaches to fabricating a mold for a nanofluidic device, using different materials and methods. One photolithographic approach that is suitable for rapid prototyping is to spin-coat and pattern SU-8, an epoxy-based negative-tone photoresist, on a silicon wafer. In this context, perhaps the simplest approach to fabricate a mold for a nanofluidic slit is to use a single layer of SU-8 with a nanoscale film thickness. However, a bilayer of SU-8, consisting of a nanoscale film of SU-8 under a microscale film of SU-8, as Fig. 5 illustrates, performs better in several aspects of the fabrication and operation of the device. In particular, Sec. 6.4 describes the punching of inlet and outlet holes from the silicone bilayer. This process can cause the hard silicone to locally deform or fracture, and a molded microscale channel is more robust against such defects than a molded nanoscale slit, which can collapse or become blocked. In addition, during device operation, microfluidic channels form connections in regions of the device where nanofluidic slits are not necessary, decreasing field gradients in these noncritical regions of the device and reducing clogging. This section describes the fabrication of a mold consisting of a bilayer of SU-8 with a nanoscale layer of SU-8 2000.5 under a microscale layer of SU-8 2025. The order is important because nanoscale features are optimally patterned in a planar film of photoresist which is conformally coated over an unpatterned substrate. Spin-coating photoresist over previously patterned features can result in radial variation in thickness across the wafer which is evident as streaking. We have spin-coated and patterned SU-8 2000.5 and SU-8 2025 on nominally clean silicon wafers, as received from the manufacturer without any additional cleaning or priming, and successfully used the resulting device molds. Table 2 presents materials for patterning a device mold in a bilayer of SU-8. Section 10 discusses approaches to patterning device molds with dimensions outside of the range enabled by these two formulations of SU-8.

### 4.1

Spin-coat a nanoscale film of SU-8 2000.5 onto a bare silicon wafer.

#### 4.1.1

Center the silicon wafer on the chuck of a spin-coater using a centering jig.

#### 4.1.2

Pipette ≈ 10 mL of SU-8 2000.5 onto the center of the silicon wafer, being careful to not create any air bubbles.

#### 4.1.3

**Fig. 6:**
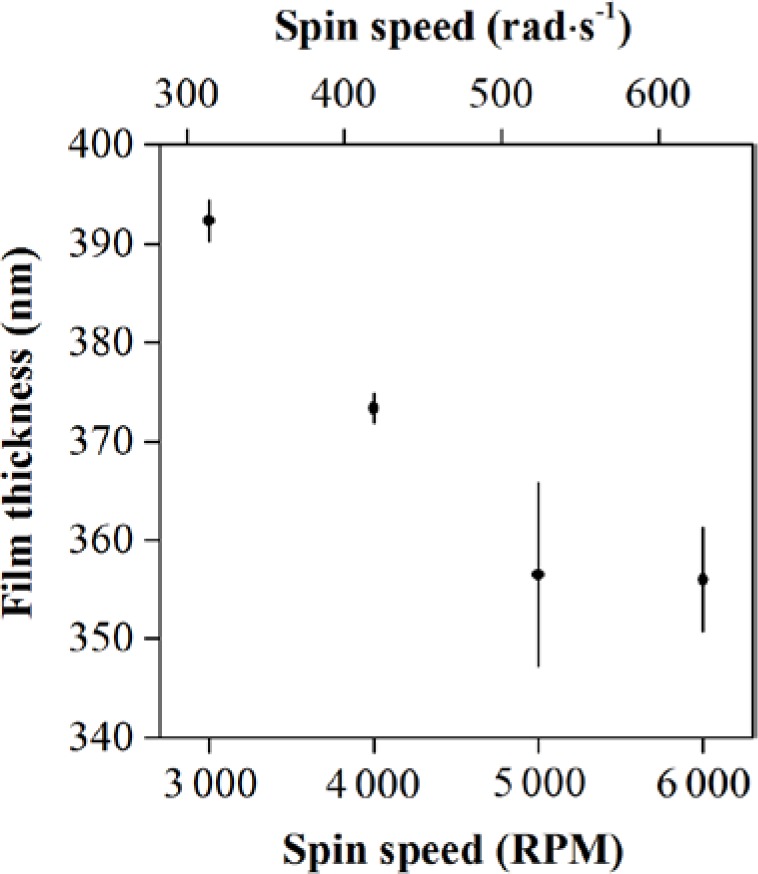
Plot showing representative data for how the nanoscale thickness of a film of SU-8 2000.5 decreases as spin speed increases above 314.2 rad·s^−1^ (3 000 RPM). Vertical bars are one standard deviation for 3, 3, 8, and 4 replicate experiments, respectively, in order of increasing spin speed.

Spin-coat following the process that Table 3 presents. The SU-8 2000.5 film thickness determines the nanofluidic channel depth, decreasing from ≈ 400 nm to ≈ 350 nm as the spin speed increases from 314.2 rad·s^−1^ (3 000 revolutions per minute [RPM]) to 628.3 rad·s^−1^ (6 000 RPM), as Fig. 6 shows.

### 4.2

Pre-exposure bake the silicon wafer with nanoscale film of SU-8 2000.5 on a hotplate at 95 °C for 2 min.

### 4.3

Perform the first photolithographic exposure of the nanoscale film of SU-8 2000.5.

#### 4.3.1

Place the silicon wafer with nanoscale film of SU-8 2000.5 along with the first photomask for patterning nanofluidic slits into the Suss MA6 contact aligner.

#### 4.3.2

Bring the silicon wafer with nanoscale film of SU-8 2000.5 and the photomask into soft contact.

#### 4.3.3

Use an i-line filter to expose the nanoscale film of SU-8 2000.5 at a wavelength of 365 nm with a dose of 80 mJ·cm^−2^.

### 4.4

Post-exposure bake the silicon wafer with exposed nanoscale film of SU-8 2000.5 on a hotplate at 95 °C for 2 min.

### 4.5

Develop the pattern in the exposed nanoscale film of SU-8 2000.5.

#### 4.5.1

Immerse the silicon wafer with exposed nanoscale film of SU-8 2000.5 in SU-8 developer for 1 min with gentle agitation.

#### 4.5.2

Rinse the silicon wafer with developed features in SU-8 2000.5 in isopropyl alcohol for 10 s and blow-dry with nitrogen.

### 4.6

Hard-bake the silicon wafer with features patterned in SU-8 2000.5 on a hotplate at 150 °C for 10 min. These patterned features adhere robustly to the substrate, and resist removal by masking tape, as Secs. 4.8 and 4.9 describe.

### 4.7

Measure the film thickness of features patterned in SU-8 2000.5 using, for example, a scanning probe surface profilometer, ellipsometer, or atomic force microscope.

### 4.8

Mask regions of the silicon wafer with lower alignment marks patterned in SU-8 2000.5 using a strip of Kapton tape with a width, for example, of ≈ 6 mm (0.25 in). This masking tape will cover the lower alignment marks during the spin-coating process that Sec. 4.9 describes. Do not cover regions of the silicon wafer where higher-level device features will be patterned, as this would prevent photolithography in these regions.

### 4.9

Spin-coat a microscale film of SU-8 2025 over the masked silicon wafer with features patterned in SU-8 2000.5.

#### 4.9.1

Center the masked silicon wafer with features patterned in SU-8 2000.5 on a spin-coater using a centering jig.

#### 4.9.2

Pipette ≈ 10 mL of SU-8 2025 onto the center of the silicon wafer with features patterned in SU-8 2000.5, being careful not to introduce any air bubbles.

#### 4.9.3

Spin coat a film of SU-8 2025 following the process that Table 3 tabulates. A spin speed of 314.2 rad·s^−1^ (3 000 RPM) gives a film thickness of > 20 µm.

#### 4.9.4

Remove the Kapton masking tape.

### 4.10

Pre-exposure bake the silicon wafer with microscale film of SU-8 2025 on a hotplate at 95 °C for 8 min.

### 4.11

Perform the second photolithographic exposure of the microscale film of SU-8 2025.

#### 4.11.1

Place the silicon wafer with the microscale film of SU-8 2025 in the Suss MA6 contact aligner, along with the second photomask for microscale features of the device.

#### 4.11.2

Align the previously patterned nanoscale fiducial marks with the microscale fiducial marks on the photomask. Make sure to align the lower and higher alignment marks, as Fig. 3 shows, for ≥ 3 pairs of alignment marks across the device array.

#### 4.11.3

Bring the silicon wafer with microscale film of SU-8 2025 and photomask into soft contact. 4.11.4. Use an i-line filter to expose the SU-8 2025 film at a wavelength of 365 nm with a dose of 160 mJ·cm^−2^.

### 4.12

Post-exposure bake the silicon wafer with exposed microscale film of SU-8 2025 on a hotplate at 95 °C for 8 min.

### 4.13

Develop the pattern in the exposed microscale film of SU-8 2025.

#### 4.13.1

Prepare two containers of SU-8 developer for an initial bulk removal of SU-8 2025, followed by a cleaning rinse.

#### 4.13.2

Immerse the silicon wafer with exposed microscale film of SU-8 2025 in the first container for 45 s with gentle agitation.

#### 4.13.3

Immerse the silicon wafer with partially developed microscale film of SU-8 2025 in the second container with gentle agitation for 30 s.

#### 4.13.4

Rinse the silicon wafer with fully developed microscale film of SU-8 2025 with isopropyl alcohol, and blow-dry with nitrogen.

### 4.14

Inspect the resulting device mold by optical microscopy. If any SU-8 2025 residue persists, then rinse the wafer again in fresh SU-8 developer for 10 s, rinse with isopropyl alcohol, and blow-dry with nitrogen.

## 5. Molding a Device

Molding processes can transfer the vertical dimensions of patterned features from a hard mold into a soft material with nanoscale fidelity [19]. Soft materials consisting primarily of polydimethylsiloxane (PDMS), also known as silicone, are useful for both pattern transfer and device function in many fluidic and optical applications. Silicone devices are typically disposable, which is consistent with the concept of rapid prototyping, and useful for avoiding contamination in biological applications. The formulation of the silicone can tune the properties and performance of the material to achieve an optimal result for a particular application. This section presents a molding process involving a bilayer of hard and soft silicone, as Fig. 1 shows. The thin film of hard silicone accepts the nanofluidic slit pattern, and inhibits the collapse of such wide and shallow channels. The elastic modulus of the hard silicone is ≈ 8 MPa [20]. The process that this article reports results in a film thickness of hard silicone of > 20 μm, which is much thicker than the nanoscale depth of a nanofluidic slit. A macroscopic slab of soft silicone functions as a backing layer to support the thin film of hard silicone. The elastic modulus of the soft silicone is ≈ 2 MPa [20]. The optimal thickness of the slab of soft silicone depends on the application of the user. As a starting point, a thickness of several millimeters provides mechanical stability and ease of handling. Silanization of both masters and glassware prior to molding ensure the subsequent removal of molded silicone. The order of the following steps of the process accounts for the long working time of soft silicone, which cures slowly, and the short working time of hard silicone, which cures quickly. Table 4 presents materials for molding devices.

### 5.1

Silanize the silicon wafer with SU-8 device molds, and a clean glass petri dish with a diameter of 150 mm, with Tridecafluoro-1,1,2,2-tetrahydrooctyl-1-trichlorosilane (TFOCS).

#### 5.1.1

Place the silicon wafer with SU-8 device molds or the petri dish in a vacuum bell jar with a volume of ≈ 3.6 L.

#### 5.1.2

Place ≈ 20 µL of TFOCS in a disposable container next to the wafer or dish.

#### 5.1.3

Pump down with a mechanical vacuum pump with a free air displacement of ≈ 50 L⋅min^−1^ for a duration of 45 s to 60 s and then vent the bell jar.

#### 5.1.4

The device mold or petri dish is now ready for use, and does not need to be re-silanized for several tens of uses.

### 5.2

Mix the soft silicone components.

#### 5.2.1

Prepare an adequate quantity of soft silicone, at the recommended ratio of pre-polymer mass to curing agent mass of 10:1. For example, prepare 66 g of Dow Corning Sylgard 184 by combining 60 g of pre-polymer with 6 g of curing agent in a large disposable container. This quantity of material, as poured over a master wafer in a petri dish with a diameter of 150 mm, will result in a backing layer of soft silicone with a thickness of ≈ 4 mm.

#### 5.2.2

Mix the pre-polymer and curing agent vigorously for ≈ 3 min. A homogeneous mixture is critical to the uniform curing of soft silicone.

### 5.3

Degas the soft silicone mixture.

#### 5.3.1

Place the soft silicone mixture in a vacuum bell jar with a volume of ≈ 3.6 L.

#### 5.3.2

Pump down with a mechanical vacuum pump with a free air displacement of ≈ 50 L⋅min^−1^.

#### 5.3.3

Take care to prevent the bubbling mixture from spilling over the edges of its container, either by firmly tapping the vacuum bell jar up and down on the benchtop of the fume hood, or by briefly venting the vacuum bell jar.

#### 5.3.4

Continue degassing the soft silicone mixture for > 10 min in order to remove all visible air bubbles from the mixture.

### 5.4

Place the silicon wafer with SU-8 device molds on a spin-coater and center the wafer on the chuck using a centering jig. Load the recipe for spin-coating hard silicone with the parameters that Table 5 presents.

### 5.5

Mix the hard silicone components.

#### 5.5.1

Remove > 2 mL of HMS-301 from the refrigerator and warm to room temperature.

#### 5.5.2

Pour 6.8 g of VDT-731 into a small disposable dish.

#### 5.5.3

Add 36 µL of platinum-divinyltetramethyldisiloxane and 100 µL of 2,4,6,8-tetramethylcyclotetrasiloxane and mix thoroughly, giving an initial mixture of hard silicone.

#### 5.5.4

Degas the initial mixture of hard silicone.

##### 5.5.4.1

Place the initial mixture of hard silicone in a vacuum bell jar with a volume of ≈ 3.6 L.

##### 5.5.4.2

Pump down the vacuum bell jar with a mechanical vacuum pump with a free air displacement of ≈ 50 L⋅min^−1^ for 2 min.

##### 5.5.4.3

Take care to prevent the initial mixture of hard silicone from spilling over the edges of its container, either by firmly tapping the vacuum bell jar up and down on the benchtop of the fume hood, or by briefly venting the vacuum bell jar. The initial mixture of hard silicone bubbles less than the mixture of soft silicone, however, due to the lower viscosity of the initial mixture of hard silicone.

#### 5.5.5

Complete the final mixture of hard silicone.

##### 5.5.5.1

Remove the initial mixture of hard silicone from the vacuum bell jar.

##### 5.5.5.2

Add 2 mL of HMS-301 and mix immediately and gently, giving a final mixture of hard silicone.

##### 5.5.5.3

This final mixture of hard silicone does not need to be degassed, and cures in < 10 min.

### 5.6

Pour the final mixture of hard silicone onto the silicon wafer with SU-8 device molds that was loaded onto the spin-coater, and spin-coat using the recipe that Table 5 presents.

### 5.7

Bake the silicon wafer with SU-8 device molds and thin film of hard silicone in a convection oven at 80 °C for 5 min.

### 5.8

Place the silicon wafer with SU-8 device molds and thin film of hard silicone facing up in a silanized glass petri dish with a diameter of 150 mm.

### 5.9

Pour over the mixture of soft silicone prepared in Secs. 5.2 and 5.3 onto the silicon wafer with SU-8 device molds and thin film of hard silicone. Pour carefully so as not to introduce any air bubbles into the device.

### 5.10

Bake the silicon wafer with SU-8 device molds, thin film of hard silicone, and thick layer of soft silicone in a convection oven at 80 °C for > 4 h.

## 6. Demolding, Cutting, and Punching a Device

Take care while demolding, cutting, and punching a device molded in a bilayer of hard and soft silicone, both to avoid breaking the silicon wafer, and to avoid fracturing the thin film of hard silicone at critical locations near the devices. It is good practice to avoid directly handling the device areas, even with gloved hands.

### 6.1

**Fig. 7:**
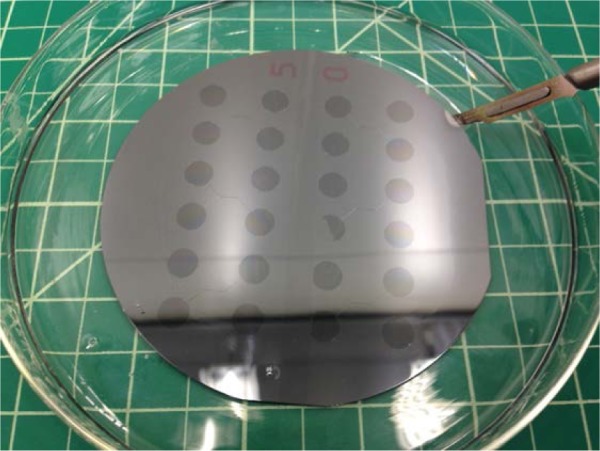
Photograph showing cutting of silicone slab around silicon wafer. The grid is ≈ 12.5 mm (0.5 in).

Remove the slab of molded silicone from the petri dish by cutting around the silicon wafer with a sharp scalpel blade, as Fig. 7 shows. Cutting with such a blade provides good manual control, but locally deforms and fractures the hard silicone.

### 6.2

**Fig. 8:**
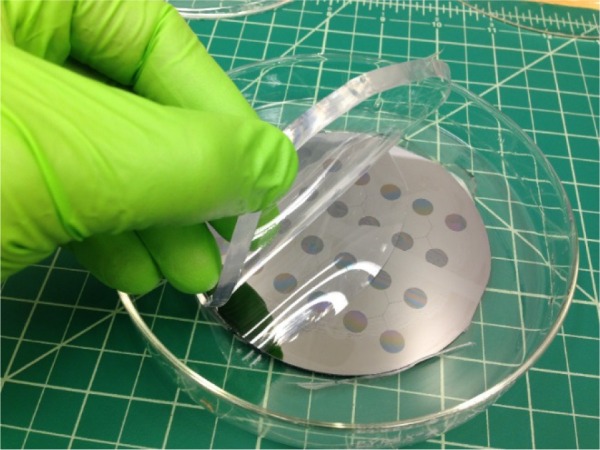
Photograph showing peeling of silicone slab from silicon mold. The grid is ≈ 12.5 mm (0.5 in).

Peel the slab of molded silicone off of the surface of the silicon wafer, as Fig. 8 shows, and lay the silicone slab on a cutting mat with the molded features facing up.

### 6.3

**Fig. 9:**
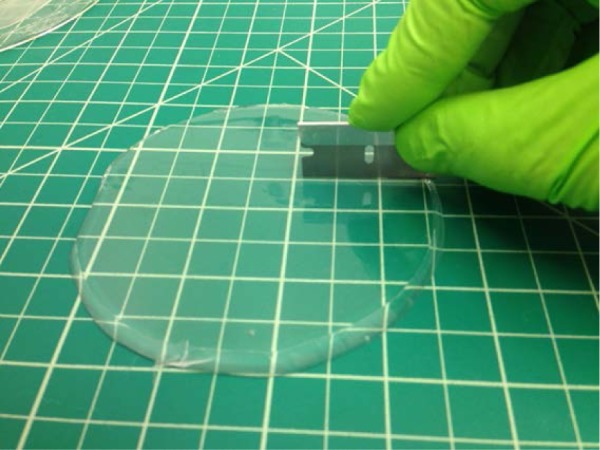
Photograph showing cutting of an individual device. The grid is ≈ 12.5 mm (0.5 in).

Cut out individual devices from the silicone slab, by vertically pressing a sharp straight-edge razor blade down into and through the silicone slab, as Fig. 9 shows. Do not horizontally shear the blade across the silicone slab, or the thin film of hard silicone can fracture and deform at critical locations.

### 6.4

**Fig. 10:**
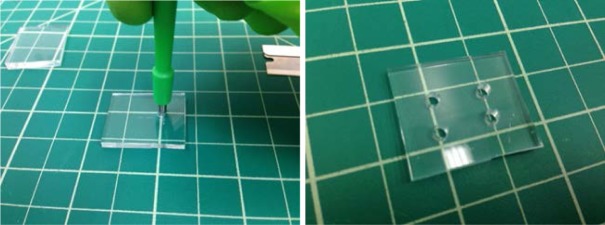
Photograph showing punching of inlets and outlets on individual device. The figure at right has a total of four. The grid is ≈ 12.5 mm (0.5 in).

Punch each inlet and outlet hole through the terminus of a microfluidic channel, using a sharp biopsy punch with a diameter of several millimeters, as Fig. 10 shows. Ensure that the holes connect to the microfluidic channels that will fill the nanofluidic slit.

### 6.5

Cover each silicone device surface with 3M Scotch Tape, folding over and adhering the end of the tape to itself, to facilitate subsequent removal of the tape from the device. Store covered devices in a clean petri dish.

### 6.6

Wash an equal number of microscope coverslips, or device substrates, serially in acetone, methanol, and deionized water, and blow-dry with nitrogen. Sec. 7 describes a cleaning process using oxygen plasma and obviating the need to bake-dry the coverslips.

### 6.7

Store covered microscope coverslips or device substrates in clean petri dishes carefully to prevent any contamination.

## 7. Bonding a Device

Device bonding involves exposing the surfaces of the hard silicone and glass coverslip, or other silica substrate, to an oxygen plasma to clean and chemically activate the surfaces. Performing this process in a clean room minimizes particulate contamination during this important step of device fabrication, while an oxygen plasma cleaner in a conventional laboratory is more accessible and economical. This section describes the use of a reactive ion etcher in a clean room to minimize particulate contamination, at the expense of additional time and cost.

### 7.1

Bring the devices and clean coverslips or other substrates into a clean room, with enough petri dishes to place each bonded device in its own dish for curing.

### 7.2

Vent the chamber of a Unaxis 790 Reactive Ion Etcher and, after removing the 3M Scotch Tape, place the devices facing patterned side up and coverslips or other substrates on the platen.

### 7.3

Evacuate the chamber and run an oxygen plasma process with the parameters that Table 6 presents.

### 7.4

**Fig. 11:**
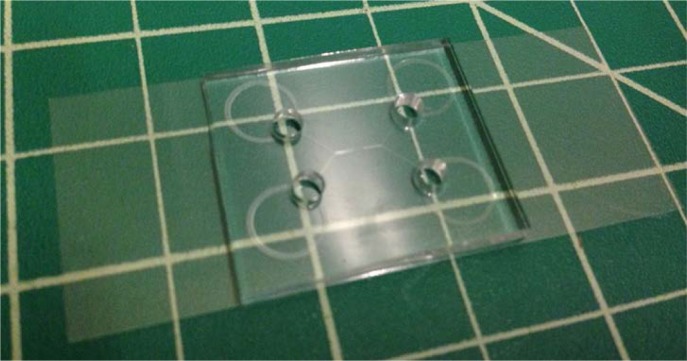
Photograph showing a final assembled device, consisting of a pair of channels of the design that Fig. 2a illustrates. For this particular device, each inlet hole and outlet hole punches through the terminus of a linear microfluidic channel, which intersects with a circular microfluidic reservoir. The grid is ≈ 12.5 mm (0.5 in).

Vent chamber and assemble devices immediately, gently pressing out visible air bubbles. Fig. 11 shows a bonded device without any visible air bubbles.

### 7.5

Place each device in its own petri dish and allow bond to cure for ≈ 10 min.

### 7.6

If the devices will not be filled with aqueous solution within a few hours, as Sec. 9 describes, then protect the devices for storage.

#### 7.6.1

Cover each silicone device surface with 3M Scotch Tape, folding over and adhering the end of the tape to itself, to facilitate subsequent removal of the tape from the device.

#### 7.6.2

Store covered devices in a clean, covered, petri dish.

## 8. Filling a Device, after Bonding

After exposure to an oxygen plasma for bonding, as Sec. 7.3 describes, devices retain hydrophilic surfaces for a few hours, greatly facilitating the filling of a device with an aqueous solution by capillarity. During the filling process, a goal is to avoid trapping bubbles of air in the device. The ideal routine for filling a device depends on the geometry of the device, but a general routine is as follows.

### 8.1

Place the device onto an optical microscope for real-time inspection.

### 8.2

Pipette an aqueous filling solution into the primary inlet reservoir. The composition of the filling solution depends on the application, but an aqueous solution with a lower viscosity will fill small fluidic channels more readily than an aqueous solution with a higher viscosity.

### 8.3

Observe the filling solution wetting the device from the primary inlet to any other inlets and outlets. 8.4. Pipette the filling solution into the other inlets and outlets.

### 8.5

Confirm that there are no air bubbles trapped in the device. If any air bubbles are trapped in the device, place the device in a petri dish with a cover, and proceed to Sec. 9.3.

## 9. Filling a Device, After Storage

After storage, as Sec. 7.6 describes, the surfaces of a device can become less hydrophilic, in which case the device will fill more easily by vacuum suction. This process makes use of the gas permeability of silicone in response to applied vacuum [21, 22].

### 9.1

Remove the 3M Scotch Tape from the silicone device and microscope coverslip or device substrate surfaces. Wash any residue from the backside of the coverslip or substrate serially with acetone, methanol, and deionized water, and blow-dry with nitrogen.

### 9.2

Pipette an aqueous filling solution into each inlet and outlet well of the device.

### 9.3

Place the entire petri dish, including its cover, into a vacuum bell jar with a volume of ≈ 3.6 L.

### 9.4

Pump down the vacuum bell jar with a mechanical vacuum pump with a free air displacement of ≈ 50 L⋅min^−1^ for 1 min.

### 9.5

Seal the vacuum bell jar and allow the device to sit under vapor pressure for 10 min.

### 9.6

Vent the vacuum bell jar and remove the petri dish with device.

## 10. Conclusion

This article has reported a comprehensive process for rapidly prototyping nanofluidic slits in silicone. This is a demanding structure to form in soft materials, due to the possibility of channel collapse at high aspect ratios of channel width to channel depth. Using this process, we have fabricated and filled nanofluidic slits with depths of less than 400 nm and aspect ratios of width to depth of greater than 250, without collapse of the channel. Other articles have reported nanofluidic slits with aspect ratios exceeding 500 [23], also fabricated using a bilayer of hard and soft silicone. The ultimate limit of the aspect ratio of the channel is not known, but this limit depends largely on the elastic modulus of the hard silicone used, which is determined by the particular formulation of this material, as well as the surface energy of the silicone and the substrate. It is possible to prepare molds for shallower or deeper fluidic channels by using other formulations of SU-8, or by patterning and etching silicon substrates. A similar fabrication process may apply to molding nanofluidic channels with a nanoscale width, using a device mold patterned with a nanoscale lateral resolution. It is possible to bond silicone nanofluidic devices to substrates other than glass coverslips, extending the utility of the approach to other applications. It may be possible to increase the strength of such bonds by annealing the bonded assembly at ≈ 100 °C. Finally, after filling the device, electrokinetic drive can load nanoscale materials into the device for measurement, depending on the properties of the materials and the solution, as well as the design of the device.

## Figures and Tables

**Table 1:** Materials for fabricating a photomask

Material	Amount
Photomask, soda lime glass, square size of 127 mm (5 in), thickness of ≈ 2.3 mm (0.09 in), pre-coated with positive tone photoresist	1
AZ 400K developer 1:3	≈ 0.1 L
Chromium Etchant 1020	≈ 0.1 L
Remover PG	≈ 0.1 L
Nanostrip	≈ 0.1 L

**Table 2:** Materials for patterning a device mold in a bilayer of SU-8

Material	Amount
Silicon wafer, diameter of 100 mm, thickness of ≈ 500 μm	1
MicroChem SU-8 2000.5 photoresist	≈ 10 mL
MicroChem SU-8 2025 photoresist	≈ 10 mL
Kapton tape, ≈ 6 mm (0.25 in) width	≈ 20 cm
SU-8 developer	≈ 0.5 L

**Table 3:** Parameters for spin-coating a nanoscale film of SU-8 2000.5

Spin speed (rad·s^−1^) (RPM)	Spin acceleration (rad·s^−2^) (RPM·s^−1^)	Time (s)
314.2 (3 000) to 628.3 (6 000)	26.18 (250)	12
314.2 (3 000) to 628.3 (6 000)	0	45
0	−26.18 (−250)	12

**Table 4:** Materials for molding devices in silicone using a petri dish with a diameter of 150 mm

Product name	Chemical name	Quantity
Gelest Platinum catalyst	Platinum-divinyltetramethyldisiloxane	36 µL
Sigma-Aldrich Modulator	2,4,6,8-tetramethylcyclotetrasiloxane	100 µL
Gelest VDT-731	(7.0 % to 8.0 % vinylmethylsiloxane)-dimethylsiloxane copolymer, trimethylsiloxy terminated	6.8 g
Gelest HMS-301^a^	(25 % to 35 % methylhydrosiloxane)-dimethylsiloxane copolymer, trimethylsiloxy terminated	2 mL
Dow Corning	Pre-polymer	60 g
Sylgard 184	Curing agent	6 g
United Chem. Tech. TFOCS	Tridecafluoro-1,1,2,2-tetrahydrooctyl-1-trichlorosilane	≈ 20 µL

a Stored at 4 °C

**Table 5:** Parameters for spin-coating a film of hard silicone with a thickness of > 20 µm

Spin speed (rad·s^−1^) (RPM)	Spin acceleration (rad·s^−2^) (RPM·s^−1^)	Time (s)
52.4 (500)	10.5 (100)	10
104.7 (1 000)	10.5 (100)	45
31.4 (300)	10.5 (100)	7
0	−3.1 (−30)	10

**Table 6:** Parameters for preparing the surfaces of hard silicone and glass for bonding

O_2_ pressure (Pa) (mTorr)	O_2_ flow rate (mL·min^−1^) (sccm)	Power (W)	Time (s)
26.7 (200)	25 (25)	100	30
